# Evaluation of inbreeding and genetic diversity in Japanese Shorthorn cattle by pedigree analysis

**DOI:** 10.1111/asj.13643

**Published:** 2021-10-13

**Authors:** Yoshinobu Uemoto, Keiichi Suzuki, Jumpei Yasuda, Sanggun Roh, Masahiro Satoh

**Affiliations:** ^1^ Graduate School of Agricultural Science Tohoku University Sendai Japan; ^2^ Iwate Prefecture Livestock Research Center, Takizawa Iwate Japan

**Keywords:** effective population size, genetic diversity, inbreeding coefficient, Japanese Shorthorn, pedigree analysis

## Abstract

The Japanese Shorthorn is a Japanese Wagyu breed maintained at a small population size. We assessed the degree of inbreeding and genetic diversity among Japanese Shorthorn cattle using pedigree analysis. We analyzed the pedigree records of registered Japanese Shorthorn born between 1980 and 2018, after evaluating the pedigree completeness. The average of the actual inbreeding coefficients increased at the same rates annually from approximately 1.5% in 1980 to 4.2% in 2018 and was higher than the expected inbreeding coefficients over time. The effective population size based on the individual coancestry rate largely decreased from 127.8 in 1980 to 82.6 in 1999, and then remained almost constant at approximately 90. Three effective numbers of ancestors decreased over time until 1995, then remained almost constant. In particular, the effective number of founder genomes (*N*_*ge*_) decreased from 43.8 in 1980 to 11.9 in 2018. The index of genetic diversity based on *N*_*ge*_ decreased from 0.99 in 1980 to 0.96 in 2018 due to genetic drift in non‐founder generations. Changes in inbreeding and genetic diversity parameters were similar between Japanese Shorthorn and other Japanese Wagyu breeds, but the magnitude of the changes was lower in the Japanese Shorthorn.

## 1 INTRODUCTION

The Japanese Shorthorn is a Japanese Wagyu breed raised in the Tohoku and Hokkaido regions of northern Japan (Motoyama et al., 2016). Japanese Shorthorn cattle were originally generated by crossing imported Western Shorthorn bulls with *Nambu‐ushi*, a breed maintained in the Iwate Prefecture. The Japanese Shorthorn has been continuously improved and was certified as a Japanese Wagyu breed in 1957 (Takayasu, 1983). Japanese Shorthorn cattle are adapted to grazing and can nurture calves because they produce abundant amounts of milk and can utilize roughage (Motoyama et al., 2016; Yamaguchi et al., 2013). In addition, Japanese Shorthorn cattle graze in a unique system known as *Natsuyama fuyusato* (mountain in summer, village in winter) (Kondo et al., 2005), in which cattle graze on pastures in summer and are fed in stalls in the winter. The most important part of the pasture period is natural mating, in which a single bull and approximately 50 cows are accommodated in a pasture plot of regional public ranches and then naturally mated (called *Makiushi*) (Mizuma & Sasaki, 1974). Thus, Japanese Shorthorn are produced and maintained with a unique breeding and production system.

The degree of beef marbling standard (BMS) and its variance are lower in Japanese Shorthorn than those in Japanese Black cattle, and carcass weight has been emphasized as a target for genetic improvement in Japanese Shorthorn cattle (MAFF, 2020). The BMS has become the most economically important trait in Japan, and Japanese Black cattle with a high degree of marbling are more suited to this trend than Japanese Shorthorn cattle. Hence, the size of the Japanese Shorthorn population has fallen to 7000–8000 individuals (NLBC, 2021). In contrast, a decline in genetic diversity is an important issue for Japanese Black cattle because of the intensive use of a few sires with a high estimated breeding value (EBV) of marbling (Honda et al., 2002, 2004; Nomura et al., 2001). Therefore, genetic selection while monitoring the genetic status of a breed is needed to sustainably use livestock materials.

The Japanese Shorthorn has a unique genetic background compared to that of other Japanese Wagyu breeds (Mannen et al., 2020; Noda et al., 2018), and the maintenance of genetic diversity is important for future genetic resources. For example, some consumers prefer lean meat, as their needs have diversified (Sasaki et al., 2017). In addition, since Japanese Shorthorn cattle graze under natural mating and seasonal breeding schemes, genetic diversity may be maintained even though the number of individuals in the population is small compared with that of other Wagyu breeds for which most progeny are produced by artificial insemination (AI). However, the current genetic status of Japanese Shorthorn cattle has not yet been determined. Pedigree analysis is a useful approach for monitoring and evaluating genetic diversity within populations based on inbreeding coefficients, effective population size, and the probabilities of gene origins. The present study aimed to clarify the degree of inbreeding and genetic diversity in Japanese Shorthorn using pedigree analysis.

## 2 MATERIALS AND METHODS

Approval of the Animal Care and Use Committee was not obtained for this study because we acquired pedigree data from an existing database.

### 2.1 Pedigree data

We analyzed the pedigree records of Japanese Shorthorn cattle that were maintained in cooperation with the Japanese Shorthorn Cattle Association of Japan and born between 1970 and 2018 to known parents. The animals born during each year served as reference populations. The total number of bulls and cows in the reference population was 1,833 and 33,850, respectively, and Table 1 shows the number of cows born in each reference population every 5 years. The pedigrees of the animals in the reference populations were traced back as far as possible to obtain a base population, and the total number of animals in the pedigree was 44,614. Ancestors with both unknown parents and those with only one known parent were regarded as founders. Table 1 shows the number of founders in each reference population every 5 years. More founders had only one known parent than two unknown parents in each period. We evaluated the parameters of pedigree completeness, inbreeding, effective population size, and genetic diversity based on pedigree data of Japanese Shorthorn cattle using R software (http://www.r-project.org).

**TABLE 1:** The number of reproductive bulls, reproductive cows, founders with both unknown parents (*N*_1_), founders with only dam known parent (*N*_2_), founders with only sire known parent (*N*_3_), and discrete generation equivalents (*g*_*e*_) in each period

Periods	Bulls	Cows	*N*_1_	*N*_2_	*N*_3_	*g*_*e*_
1970–1974	198	369	210	1	407	2.2
1975–1979	358	2750	1035	12	1341	2.5
1980–1984	337	9214	1705	24	3124	3.1
1985–1989	311	8662	1431	17	2789	3.8
1990–1994	163	4181	861	7	1804	4.4
1995–1999	130	2508	539	3	1265	5.1
2000–2004	85	1624	382	2	951	5.7
2005–2009	104	1782	346	1	853	6.3
2010–2014	89	1485	316	1	776	6.9
2015–2018	58	1275	281	1	701	7.5

### 2.2 Pedigree completeness and generation intervals

The completeness and depth of the pedigree were evaluated before analyzing inbreeding, effective population size, and genetic diversity in our population. The pedigree completeness index (PCI) and discrete generation equivalents (*g*_*e*_) were used as indicators. PCI is an important indicator of pedigree quality for inbreeding estimation, and its value is 1 when all ancestors are known in the paternal and maternal lines traced back to *d* generations. We calculated the PCI (MacCluer et al., 1983; Sørensen et al., 2005) for each reference population as follows:

$$\mathrm{PCI}=\frac{2I_{p,d}I_{m,d}}{I_{p,d}+I_{m,d}},$$

where $I_{p,d}$ and $I_{m,d}$ are the proportions of known ancestors averaged over *d* generations in the paternal (*p*) and maternal (*m*) lines, respectively. The proportion of known ancestors was calculated as follows:

$$I_{i,d}=\frac{1}{d}\sum_{j=1}^{d}u_{i,j},$$

where $u_{i,j}$ is the proportion of known ancestors at generation *j* in line *i*. The PCI over three and five previous generations in the pedigree were calculated and are referred to as PCI3 and PCI5, respectively. The informative reference population, which we defined as PCI3 > 0.75 and PCI5 > 0.50, was applied to evaluate inbreeding, effective population size, and genetic diversity.

The complete generation equivalent (CGE) is defined as the sum of the proportion of known ancestors over all generations traced (Maignel et al., 1996) and is calculated according to Boichard (2002) as follows:

$$\mathrm{CGE}=\sum_{i=1}^{n}\frac{1}{2^{g_{i}}},$$

where *n* is the total number of ancestors and *g*_*i*_ is the number of generations between ancestor *i* and the animal. The CGE is *d* when all ancestors are known in the previous *d* generations. The *g*_*e*_ is the expected number of generations from the base population to the reference population under discrete generation (Woolliams & Mäntysaari, 1995) and was calculated for each reference population as follows:

$$g_{e}=\bar{\mathrm{CGE}}=\frac{1}{N}\sum_{j=1}^{N}\sum_{i=1}^{n_{j}}\frac{1}{2^{g_{\mathrm{ij}}}},$$

where *n*_*j*_ is the total number of ancestors of animal *j* in the reference population, *g*_*ij*_ is the number of generations between animal *j* and its ancestor *i*, and *N* is the number of animals in the reference population.

The average generation interval (*L*) in each period was calculated as

$$L=\frac{L_{\mathrm{ss}}+L_{\mathrm{sd}}+L_{\mathrm{ds}}+L_{\mathrm{dd}}}{4},$$

where *L*_*ss*_, *L*_*sd*_, *L*_*ds*_, and *L*_*dd*_ are the generation intervals of four gametic pathways: sire to son, sire to daughter, dam to son, and dam to daughter, respectively.

### 2.3 Inbreeding and effective population size

We calculated the *F* statistics (*F*_*IT*_, *F*_*ST*_, and *F*_*IS*_), in which *F*_*IT*_ is the average inbreeding coefficient of the reference population, and the inbreeding coefficient of each individual in the pedigree was calculated according to Meuwissen and Luo (1992). The *F*_*ST*_ is the inbreeding coefficient expected under random mating and is calculated as the average coancestry between the sires and dams of the reference population. The coancestry between animals *i* and *j* (*C*_*ij*_) was calculated from the relationship of $2C_{\mathrm{ij}}=a_{\mathrm{ij}}$, where *a*_*ij*_ is the element of the additive relationship matrix **A** and an additive relationship coefficient between animals *i* and *j* (Lynch & Walsh, 1998). The *F*_*IS*_ was calculated according to Wright (1951) as follows:

$$F_{\text{IS}}=\frac{F_{\mathrm{IT}}-F_{\mathrm{ST}}}{1-F_{\mathrm{ST}}},$$

where *F*_*IT*_ indicates actual inbreeding, and *F*_*ST*_ and *F*_*IS*_ indicate expected inbreeding under random mating and deviation of the actual mating from randomness, respectively.

Two types of effective population sizes (*N*_*eFi*_ and *N*_*eCi*_) were calculated based on the individual inbreeding rate ( $∆F_{i}$) (Gutiérrez et al., 2009) and individual coancestry rate ( $∆C_{\mathrm{ij}}$) (Cervantes et al., 2011), respectively, as

$$∆F_{i}=1-\sqrt[{q_{i}-1}]{1-F_{i}},$$

$$∆C_{\mathrm{ij}}=1-\sqrt[{\left(q_{i}+q_{j}\right)/2}]{1-C_{\mathrm{ij}}},$$

where *F*_*i*_ is the inbreeding coefficient of animal *i*, *C*_*ij*_ is the coancestry between animals *i* and *j*, and *q*_*i*_ and *q*_*j*_ are their respective CGEs. Then *N*_*eFi*_ and *N*_*eCi*_ were calculated for each reference population using the average values of $∆F_{i}$ ( $\bar{∆F}$) and $∆C_{\mathrm{ij}}$ ( $\bar{∆C}$) as

$$N_{\mathrm{eFi}}=\frac{1}{2\bar{∆F}},$$

$$N_{\mathrm{eCi}}=\frac{1}{2\bar{∆C}}.$$

### 2.4 Genetic diversity

Three effective numbers of ancestors were estimated to assess genetic diversity and clarify the causes of their loss. The effective number of founders (*N*_*ef*_) is an indicator of founder contributions to the population and is defined as the number of equally contributing founders (Lacy, 1989). We calculated *N*_*ef*_ based on the algorithm described by Sargolzaei et al. (2006) as follows:

$$N_{\mathrm{ef}}=\frac{1}{\sum_{i=1}^{N_{0}}{\mathrm{EC}}_{i}^{2}},$$

where *N*_0_ is the actual number of founders that appeared in a pedigree and *EC*_*i*_ is the expected contribution of founder *i* to the reference population. *EC*_*i*_ is calculated as ${\mathrm{EC}}_{i}={\mathrm{EC}}_{0,i}$ if founders are ancestors with two unknown parents and ${\mathrm{EC}}_{i}={\mathrm{EC}}_{0,i}/2$ if founders are ancestors with one known parent. Thus, ${\mathrm{EC}}_{0,i}$ was calculated as follows:

$${\mathrm{EC}}_{0,i}=\frac{\sum_{j=1}^{N}t_{\mathrm{ji}}}{N},$$

where *N* is the number of animals in the reference population and *t*_*ji*_ is an element of the lower triangular matrix **T** that represents the fraction of the gene that animal *j* inherited from founder *i*. Matrix **T** is a component of the factorization of the additive relationship matrix $A=\mathrm{TD}T^{\prime }$ (Henderson, 1976), where the diagonal matrix **D** contains within‐family segregation variances.

Only the loss of genetic diversity due to unequal founder contributions to the reference population is explained by *N*_*ef*_. Genetic diversity can also be lost due to genetic drift in non‐founder generations, even if the founders contributed equally to the reference population (Lacy, 1989). Thus, the effective number of founder genomes (*N*_*ge*_), also referred to as the founder genome equivalent, is defined as the number of equally contributing founders without loss of founder alleles in the reference population. Thus, *N*_*ge*_ is calculated as follows:

$$N_{\mathrm{ge}}=\frac{1}{\sum_{i=1}^{N}\sum_{j=1}^{N}\frac{a_{\mathrm{ij}}}{N^{2}}},$$

where $a_{\mathrm{ij}}$ is an additive relationship coefficient between animals *i* and *j* (Caballero & Toro, 2000; Honda et al., 2004).

The loss of genetic diversity because of unequal founder contributions and genetic drift accumulated in the non‐founder generations is accounted for by *N*_*ge*_. In contrast, the effective number of non‐founders (*N*_*enf*_) accounts for the loss of genetic diversity due to the latter effect. Hence, *N*_*enf*_ is calculated according to Caballero and Toro (2000) as follows:

$$\frac{1}{N_{\mathrm{ge}}}=\frac{1}{N_{\mathrm{ef}}}+\frac{1}{N_{\mathrm{enf}}}.$$

The loss of genetic diversity can be derived from *N*_*ef*_, *N*_*ge*_, and *N*_*enf*_. We calculated the coefficients of genetic diversity (GD* and *GD*) in the reference population and expressed them as predicted heterozygosity (Caballero & Toro, 2000; Lacy, 1989; Nei, 1973). GD* accounts for the loss of genetic diversity due to an unequal founder contribution and was calculated as follows:

$${\mathrm{GD}}^{*}=1-\frac{1}{2N_{\mathrm{ef}}}.$$

In contrast, *GD* accounts for the loss of genetic diversity because of unequal founder contributions and genetic drift accumulated in non‐founder generations, and was calculated as

$$\mathrm{GD}=1-\frac{1}{2N_{\mathrm{ge}}}.$$

## 3 RESULTS

### 3.1 Pedigree completeness and generation intervals

**FIGURE 1:**
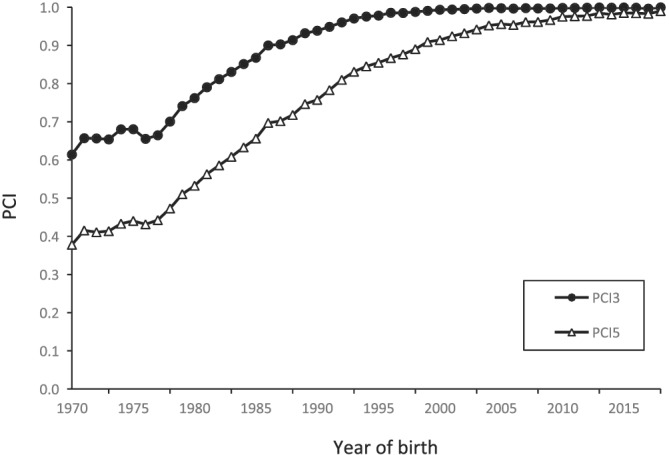
Average pedigree completeness index over three (PCI3) and five (PCI5) generations per reference population

Table 1 shows that 35,683 calves were born to two known parents between 1970 and 2018. We evaluated the depth of the pedigree by calculating the values for each reference population (Table 1). The *g*_*e*_ increased at roughly the same rate after 1975, and the most recent value was 7.5. We evaluated the completeness of the pedigree for each reference population by calculating PCI3 and PCI5 spanning three and five generations in the pedigree (Figure 1). The informative reference population, which we defined as PCI3 > 0.75 and PCI5 > 0.5, was the reference population between 1980 and 2018 that we included in the pedigree analysis. This reference population contained 32,008 calves born between 1980 and 2018.

Table 2 shows the generation intervals of the four gametic pathways and the average generation interval for each period of the reference population. The average generation intervals slightly increased from 6.0 for 1980–1984 to 7.5 for 2015–2018. The values of *L*_*sd*_, *L*_*ds*_, and *L*_*dd*_ increased slightly over time. In contrast, the value of *L*_*ss*_ differed between periods, and a specific tendency was not evident. The *L*_*ss*_ values in 1980–1984 and 2015–2018 were both 7.5.

**TABLE 2:** Generation intervals of four gametic pathways and the average generation interval (L) in each period

Periods	*L*_*ss*_	*L*_*sd*_	*L*_*ds*_	*L*_*dd*_	*L*
1980–1984	7.5	6.3	5.8	4.5	6.0
1985–1989	7.1	6.3	5.9	5.5	6.2
1990–1994	7.4	6.6	6.2	6.3	6.6
1995–1999	9.6	7.2	6.3	6.7	7.5
2000–2004	7.4	6.2	6.9	7.1	6.9
2005–2009	10.6	7.9	6.3	7.2	8.0
2010–2014	7.9	8.3	6.6	6.5	7.3
2015–2018	7.5	9.4	6.6	6.6	7.5

*Note: L*_*ss*_: sire to son; *L*_*sd*_: sire to daughter; *L*_*ds*_: dam to son; *L*_*dd*_: dam to daughter.

### 3.2 Inbreeding and effective population size

**FIGURE 2:**
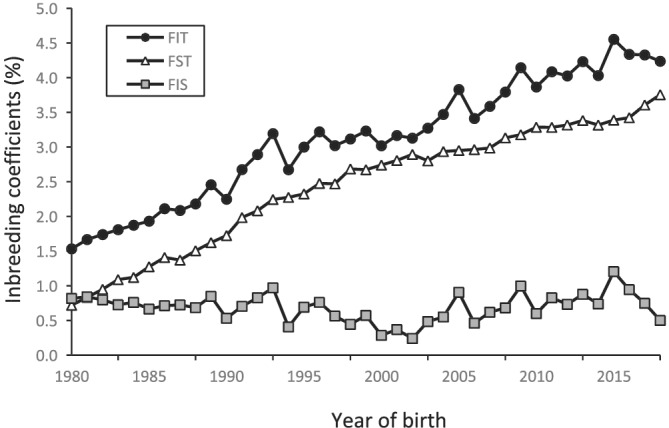
Changes in *F* statistics between 1980 and 2018

Figure 2 shows the *F* statistics (*F*_*IT*_, *F*_*ST*_, and *F*_*IS*_) for each reference population between 1980 and 2018. The *F*_*IT*_ increased at the same rate, from approximately 1.5% in 1980 to 4.2% in 2018. The *F*_*ST*_ also increased at the same rate from approximately 0.7% in 1980 to 3.8% in 2018, and *F*_*ST*_ was lower than *F*_*IT*_ in all reference populations. The *F*_*IS*_ was positive in all reference populations and almost constant at approximately 0.5% over time. Thus, the population was genetically subdivided in all reference populations (*F*_*IT*_ > *F*_*ST*_, *F*_*IS*_ > 0).

**FIGURE 3:**
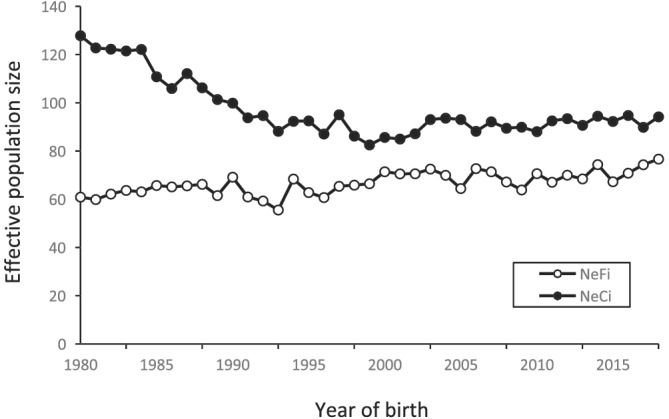
Changes in effective population size based on the individual inbreeding rate (*N*_*eFi*_) and individual coancestry rate (*N*_*eCi*_) between 1980 and 2018

Figure 3 shows two types of effective population sizes (*N*_*eFi*_ and *N*_*eCi*_) for each reference population between 1980 and 2018. *N*_*eFi*_ remained almost constant over time and ranged from 60 to 80. In contrast, *N*_*eCi*_ largely decreased from 127.8 in 1980 to 82.6 in 1999, and then remained almost constant at approximately 90. The harmonic means of *N*_*eFi*_ and *N*_*eCi*_ for four periods (1980–1989, 1990–1999, 2000–2009, and 2010–2018) were calculated and are shown in Table 3. The *N*_*eFi*_ increased slightly from 63.3 in 1980–1989 to 70.9 in 2010–2018. Conversely, *N*_*eCi*_ decreased from 114.7 in 1980–1989, and then remained almost constant at approximately 90.

**TABLE 3:** The harmonic means of effective population size based on individual inbreeding rate (*Ne*_*Fi*_) and individual coancestry rate (*Ne*_*Ci*_) in each period

Periods	*N*_*eFi*_	*N*_*eCi*_
1980–1989	63.3	114.7
1990–1999	63.2	91.0
2000–2009	69.3	89.6
2010–2018	70.9	92.2

### 3.3 Genetic diversity

**FIGURE 4:**
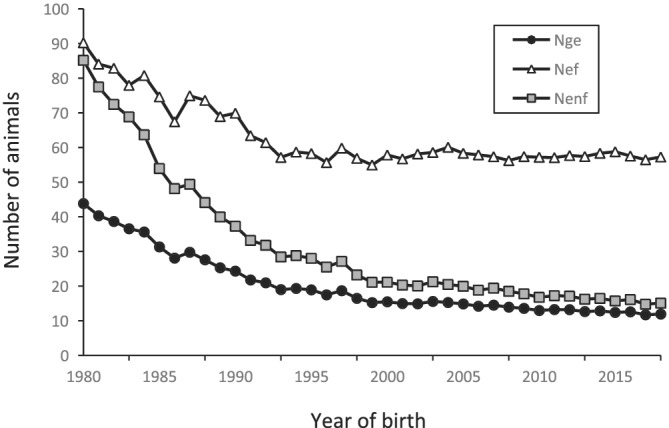
Changes in the effective numbers of founders (*N*_*ef*_), founder genomes (*N*_*ge*_), and non‐founders (*N*_*enf*_) between 1980 and 2018

**FIGURE 5:**
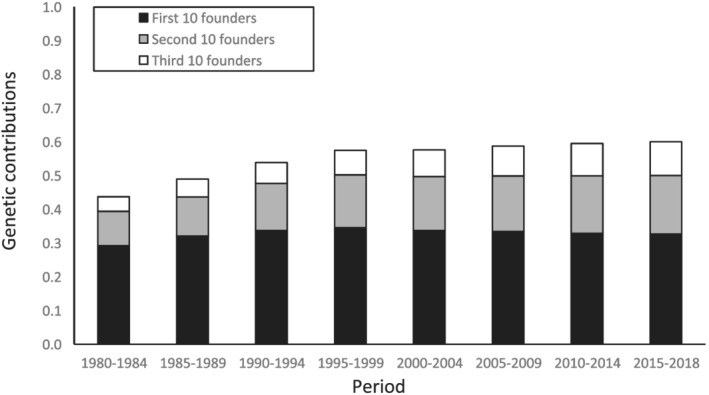
Cumulative genetic contributions for the top 30 represented founders in each period

**FIGURE 6:**
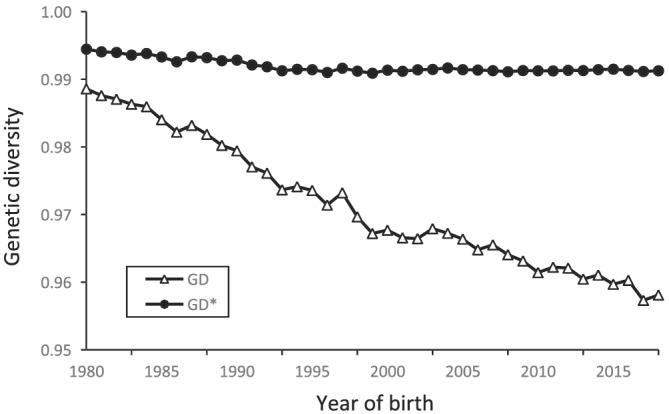
Changes in genetic diversity between 1980 and 2018. Genetic diversity (*GD*) accounts for effects of unequal founder contributions and genetic drift, whereas GD* accounts for unequal founder contributions

Figure 4 shows that three effective numbers of ancestors (*N*_*ef*_, *N*_*ge*_, and *N*_*enf*_) decreased over time until 1995, then remained almost constant at approximately 60 in *N*_*ef*_ and 20 in *N*_*ge*_ and *N*_*enf*_. The *N*_*enf*_ largely decreased from 85.1 in 1980 to 28.0 in 1995. The most recent *N*_*ef*_, *N*_*ge*_, and *N*_*enf*_ were 57.2, 11.9, and 15.1, respectively. Figure 5 summarizes changes in the genetic contribution of the founders and shows the cumulative values of the genetic contributions of the first, second, and third sets of 10 founders that were the most represented in each period. The cumulative values of the top 30 contributing founders increased from 0.44 between 1980 and 1984 to 0.57 between 1995 and 1999, then remained almost constant at approximately 0.60 by the year 2000. The genetic contribution of the first 10 founders remained almost constant at approximately 0.3. Figure 6 shows changes in the coefficients of genetic diversity (GD* and *GD*) in the reference population. The GD* remained almost constant at 0.99, whereas *GD* decreased from 0.99 in 1980 to 0.96 in 2018. The *GD* accounts for the effects of unequal genetic contributions of founders and genetic drift, whereas GD* accounts for only unequal genetic contributions of founders. These results indicated that the effect of random genetic drift mainly caused a reduction in genetic diversity over generations of non‐founders compared with the unequal contributions of founders.

## 4 DISCUSSION

### 4.1 Importance of monitoring genetic diversity

Native livestock breeds can provide useful genetic resources for adaptation to different environments and economic demands because of their unique genes. A recent survey of genetic variability in cattle using DNA markers revealed that the genetic background of Japanese Shorthorn is unique. Haplogroups in domestic cattle can be characterized worldwide by evaluating variations in mitochondrial DNA (mtDNA). The mtDNA haplogroup P was once the most common haplogroup in European aurochs according to surveys of ancient DNA (Edwards et al., 2007), but it has not been detected in modern cattle in Europe or in other Japanese Wagyu breeds. However, the mtDNA haplogroup P has been identified at a high frequency in Japanese Shorthorn cattle, indicating that the propagation root of *Nambu‐ushi* differs from that of other Japanese native cattle (Mannen et al., 2020; Noda et al., 2018). Uemoto et al. (2017) and Sasago et al. (2018) also identified the allele frequencies of beef palatability genes in four breeds (Japanese Black, Japanese Brown, Japanese Shorthorn, and Holstein), and those of the Japanese Shorthorn were completely different from those of the other three breeds. Thus, the genetic background of the Japanese Shorthorn is unique among Japanese Wagyu breeds, and the native livestock breed must be conserved.

Several factors can accelerate the reduction of minor breeds, and the main cause is associated with economic pressure, meaning the intensive use of a few highly productive breeds in livestock production. The liberalization of beef import restrictions and the genetic evaluation of carcass traits in animal models with the best linear unbiased prediction began in 1991. In addition, the intensive application of AI has accelerated since the 1990s. These trends have led to an increased dependence on the production of Japanese Black cattle, while the Japanese Shorthorn cattle population rapidly declined. However, the decline in genetic diversity is a serious problem even in the Japanese Black population because of the intensive use of a few sires with a high EBV due to abundant marbling (Honda et al., 2002, 2004; Nomura et al., 2001). Therefore, genetic selection should be performed while monitoring the genetic status of a breed to sustainably use livestock materials.

### 4.2 Comparison of Japanese Shorthorn with other Japanese Wagyu breeds

Among the four Japanese Wagyu breeds, Japanese Black and Brown are the top two major breeds, with populations of approximately 1,700,000 and 22,000, respectively (NLBC, 2021). The degree of inbreeding and genetic diversity in Japanese Wagyu breeds have been determined by comprehensive pedigree analyses of Japanese Black (Honda et al., 2002, 2004; Nomura et al., 2001, 2005) and Japanese Brown (Honda et al., 2006) breeds.

Regarding the generation interval, the average generation interval of Japanese Black and the Kumamoto and Kouchi sub‐breeds of Japanese Brown are 10.0 in 1997, 9.4 in 1991–2000, and 10.4 in 1991–2000, respectively (Honda et al., 2006; Nomura et al., 2001). The average generation interval of Japanese Shorthorn was lower than that of the other two breeds (7.5 in 1995–1999). The main reason is that the *L*_*ss*_ of Japanese Shorthorn was approximately one‐half lower than that of the other two breeds. The Japanese Shorthorn bulls are mainly distributed to regional public ranches for natural mating after performance testing, although the bulls of the other two breeds are distributed after progeny testing.

Regarding inbreeding, the *F*_*IT*_ of Japanese Black and the Kumamoto and Kouchi sub‐breeds of Japanese Brown in 2000 are 6.0%, 7.1%, and 8.8%, respectively (Honda et al., 2006; Nomura et al., 2005). The trends of *F* statistics over time in these breeds are similar and clearly reflect a change in the breeding structure (Honda et al., 2006; Nomura et al., 2001). Local differentiation among sires has rapidly diminished, and a genetically subdivided structure disappeared in the late 1990s. We showed that the *F*_*IT*_ was 3.0% in 2000 and 4.2% in 2018, indicating that less inbreeding has occurred in Japanese Shorthorn than in the other two breeds, even in the current population. In addition, the trends of *F* statistics over time differed from those of Japanese Black and Japanese Brown. The *F*_*ST*_ was lower than the *F*_*IT*_ in all reference populations, and the structure was genetically subdivided in all reference populations in Japanese Shorthorn.

The effective population size of Japanese Black (Nomura et al., 2001) and Japanese Brown (Honda et al., 2006) have been reported. The effective population size of Japanese Black and the Kumamoto and Kouchi sub‐breeds of Japanese Brown consistently reduced to 17.2, 25.5, and 6.0, respectively, during the 1990s. In contrast, the effective population size of Japanese Shorthorn was much higher than that of the other two breeds in the past and current populations. The effective population sizes of the two breeds were evaluated based on the increase in *F*_*ST*_ per generation (Falconer & Mackay, 1996; Nomura et al., 2001). In contrast, we evaluated the effective population size based on individual identity by descent (IBD) probability (*N*_*eFi*_ and *N*_*eCi*_), because a decrease in *F*_*ST*_ per generation occurred in some periods in our population, leading to a negative value of effective population size (Leroy et al., 2013). An individual increase in inbreeding is strongly affected by the population structure, and an increase in coancestry is only slightly affected by the structure (Leroy et al., 2013). Thus, *N*_*eCi*_ was higher than *N*_*eFi*_ in all reference populations, and *N*_*eCi*_ was more appropriate for calculating the effective population size of the Japanese Shorthorn, which is composed of a subdivided population. In this population, *N*_*eCi*_ is 92.2 in the current population, which suggests that high genetic diversity is maintained. As for the Japanese Shorthorn, the number of prefectures supplying running bulls for natural mating decreased during the 1990s, and most of the supply has originated from the Iwate Prefecture since the 2000s. However, *N*_*eCi*_ was maintained at approximately 90 in 1990–1999, 2000–2009, and 2010–2018, suggesting that the decrease in the number of prefectures supplying running bulls did not have a strong effect on the effective population size of Japanese Shorthorn.

The effective numbers of ancestors, *N*_*ef*_, *N*_*ge*_, and *N*_*enf*_, and two coefficients of genetic diversity (GD* and *GD*) in Japanese Black (Honda et al., 2004) and Japanese Brown (Honda et al., 2006) have been reported. The *N*_*ef*_, *N*_*ge*_, and *N*_*enf*_ were reduced to 50.3, 7.3, and 8.5 in the Japanese Black, 74.4, 4.9, and 5.3 in the Kumamoto sub‐breed of Japanese Brown, and 79.4, 3.9, and 4.1 in the Kouchi sub‐breed of Japanese Brown, respectively, by 2000. The *N*_*ef*_ was much larger than *N*_*enf*_, and *N*_*ef*_ was close to *N*_*ge*_ in both breeds. Changes in trends among the three effective numbers of ancestors were similar among Japanese Shorthorn cattle, but the *N*_*ge*_ and *N*_*enf*_ in the current population were still higher than those in both breeds. In addition, the *GD* in Japanese Black and Japanese Brown was reduced to 0.93 and <0.90, respectively, in 2000, while the trends of GD* were almost constant at 0.99 in both breeds. Trends in the two coefficients of genetic diversity were similar in Japanese Shorthorn, but the *GD* in the current population was still higher than that in both breeds. These results suggest that random genetic drift accumulation in non‐founder generations is a more serious cause of the reduction in genetic diversity than the unequal contributions of founders in the three breeds. In addition, the low value of *N*_*ge*_ indicates extremely limited genetic diversity in the current Japanese Brown population (Honda et al., 2006). The tendency to lose genetic diversity was similar in Japanese Shorthorn, but the reduction rate of the genetic diversity was much lower than that of the other two breeds.

### 4.3 Characteristics of Japanese Shorthorn cattle

Changes in the degree of inbreeding and the genetic diversity of the Japanese Shorthorn were similar to those of the other Japanese Wagyu breeds, which means that the inbreeding coefficients increased while genetic diversity decreased over time. However, the magnitude of the change in Japanese Shorthorn was much smaller than that in the other Japanese Wagyu breeds. There are two possible explanations for this maintained genetic diversity. First, a few specific sires were not used intensively. The average BMS of Japanese Shorthorn is 2.1, its phenotypic variance is low, and the estimated heritability is 0.14 (Sato et al., 2013). Thus, BMS is not the main trait for genetic improvement in Japanese Shorthorn, and the intensive use of a few sires with an EBV of marbling has not been implemented, although BMS is included as a selection index in some regions to improve meat quality, such as firmness of beef. Another reason is the natural mating system of Japanese Shorthorn. Although natural mating has a higher conception rate than that of AI, more running bulls are needed. Thus, the widespread application of AI leads to a lower cost of running bulls for mating programs and more genetic improvement (Vishwanath, 2003). However, most Japanese Shorthorn cattle are still produced by natural mating, and a number of running bulls used for breeding programs leads to the maintenance of genetic diversity.

Regarding the impact of natural mating on genetic diversity, natural mating requires more running bulls. Thus, the *F*_*IT*_ and *F*_*ST*_ of Japanese Shorthorn were low at 4.2% and 3.8%, respectively, in the current population, and higher genetic diversity was maintained compared with that in other breeds generated by AI. Conversely, local differentiation has been retained among the dams used in each regional public ranch, and each running bull is distributed to regional public ranches by accounting for the inbreeding coefficient. Thus, the population has been genetically subdivided in the past and current populations of Japanese Shorthorn. Another problem with the natural mating system is that it is difficult to completely manage pedigree information. Here, we extracted information about the reference population based on PCI, and pedigree analysis was then applied to the informative reference population. However, incomplete pedigree information might also be involved in maintaining genetic diversity in Japanese Shorthorn.

Although the degree of BMS is lower in Japanese Shorthorn than that in Japanese Black cattle, Japanese Shorthorn cattle have adapted well to a harsh climate; they are also suited to grazing and can nurture calves due to abundant milk production. The Japanese Shorthorn has a unique genetic background compared with that of other Japanese Wagyu breeds. Consumer demand for high‐quality lean meat might increase as a consequence of the aging society and health consciousness. Thus, Japanese Shorthorn genes and their gene combinations may serve as breeding materials. Although the current breeding program can maintain genetic diversity in the Japanese Shorthorn, accumulated inbreeding and the loss of genetic diversity are potentially problematic because of the decreasing number of registered bulls over time. Therefore, it is necessary to perform genetic selection while monitoring the genetic status and managing selection and mating if economic trends change for the intensive use of a few specific sires.

## 5 CONCLUSION

This study evaluated the current genetic status of the Japanese Shorthorn by clarifying the degree of inbreeding and genetic diversity using pedigree analysis. Changes in inbreeding and genetic diversity parameters were similar between Japanese Shorthorn and other Japanese Wagyu breeds, which means that the inbreeding coefficients increased and genetic diversity decreased over time. However, the magnitude of the change in Japanese Shorthorn was much smaller than that in other Japanese Wagyu breeds. The current breeding program of Japanese Shorthorn can maintain genetic diversity, but accumulated inbreeding and the loss of genetic diversity are potentially problematic, and monitoring the genetic status is necessary.

## CONFLICTS OF INTEREST

The authors have no conflicts of interest associated with this manuscript to declare.
